# Complete Genome Sequence of a Coxsackievirus A16 Strain, Isolated from a Fatal Case in Shenzhen, Southern China, in 2014

**DOI:** 10.1128/genomeA.00391-15

**Published:** 2015-04-30

**Authors:** Long Chen, Hong Yang, Qian-Jin Feng, Xiang-Jie Yao, Hai-Long Zhang, Ren-Li Zhang, Ya-Qing He

**Affiliations:** a Major Infectious Disease Control Key Laboratory and Shenzhen Public Service Platform of Pathogenic Microorganisms Repository, Shenzhen Center for Disease Control and Prevention, Shenzhen, People’s Republic of China; b Department of Health Statistics and Epidemiology, College of Public Health, Sun Yat-sen University, Guangzhou, People’s Republic of China

## Abstract

We determined the complete genome sequence of a coxsackievirus A16 strain (CVA16/SZ29/CHN/2014) from a fatal case in Shenzhen, southern China, in 2014. The strain was assigned to subgenotype B1b based on phylogenetic analysis of the VP1 gene.

## GENOME ANNOUNCEMENT

Coxsackievirus A16 (CVA16), a member of the enterovirus A species of the family *Picornaviridae,* is a common causative agent of hand, foot, and mouth disease (HFMD) in children and infants (1). Enteroviruses are a group of naked, positive, single-stranded RNA viruses, and their genomes consist of a 5′ untranslated region (UTR), a structural polypeptide P1, nonstructural polypeptides P2 and P3, and a 3′ UTR (2). CVA16 can be phylogenetically classified into two main genogroups (A and B) and five genotypes (A, B1a to B1c, and B2) based on VP1 genes (3). Worldwide epidemiological studies of HFMD have shown that severe HFMD was mainly associated with enterovirus 71 infection, whereas CVA16 and a number of other enterovirus species A serotypes usually cause mild self-limiting infections (4, 5).

On 13 May 2014, a 2.7-year-old boy manifested symptoms of hand, foot, and mouth disease accompanied by severe vomiting, lethargy, muscle twitching, and aseptic encephalitis. The boy died in a few days after ineffective treatment. The fecal specimen from this case was detected positive for CVA16 by real-time reverse transcription (RT)-PCR and propagated in rhabdomyosarcoma (RD) cell lines at the Department of Microbiology, Shenzhen Center for Disease Control and Prevention, Shenzhen, China. This is the first case of death caused by CVA16 in Shenzhen since sentinel surveillance for HFMD began in 2008.

We designed a pair of universal primers EVA-F30 (5′-TTAAAACAGCCTGTGGGTTGTACCCACCCA-3′) and EVA-R36 (5′-GCTATTCTGGTTATAACAAATTTACCCCCACCAGTC-3′) to amplify the whole genome of CVA16 strain using a PrimeScript One Step RT-PCR kit version 2 (TaKaRa, Japan). The amplification mixture was run under the following conditions: reverse transcription for 30 min at 50°C, initial denaturation for 2 min at 94°C, 45 cycles of 30 s at 94°C, 30 s at 55°C, 8 min at 72°C, and 10 min at 72°C. The amplified DNA product was sequenced by a commercial corporation (TaKaRa, Japan) using a primer-walking method. Contigs were assembled using Sequencer version 4.9. The raw genome sequence was edited using BioEdit version 7.2.5 before submission to GenBank. Nucleic acid and amino acid sequence alignments were done with BioEdit version 7.2.5. Phylogenetic analysis was performed using MEGA version 5 (6).

The full-length genome of the CVA16 strain CVA16/SZ29/CHN/2014 was composed of 7,410 nucleotides (nt), excluding the poly(A) tail. The 5′ UTR was found to be 746 nt, followed by an open reading frame (ORF) encoding the structural protein P1 (2,586 nt), the nonstructural proteins P2 (1,734 nt) and P3 (2,259 nt), and the 3′ UTR (82 nt). The contents of A, C, G, and U were 27.73%, 23.29%, 23.82%, and 25.16%, respectively, with G+C contents of 47.11%. The genome sequence of CVA16/SZ29/CHN/2014 was found to be closely related to the Chinese CVA16 strain BJ08/07 (GenBank accession no. JX068833) with 98.06% nucleotide identity by a BLAST research. The strain CVA16/SZ29/CHN/2014 was assigned to subgenotype B1b based on phylogenetic analysis of the VP1 gene.

### Nucleotide sequence accession number. 

The full-length sequence of CVA16/SZ29/CHN/2014 has been deposited in GenBank under the accession number KM215267.
